# Social differences in cause-specific infant mortality at the dawn of the demographic transition: New insights from German church records

**DOI:** 10.1007/s11111-025-00483-w

**Published:** 2025-03-13

**Authors:** Michael Mühlichen, Gabriele Doblhammer

**Affiliations:** 1 Federal Institute for Population Research (BiB), Wiesbaden, Germany; 2 Institute for Sociology and Demography, Department of Economics and Social Sciences, University of Rostock, Rostock, Germany; 3 Demographic Studies, German Centre for Neurodegenerative Diseases (DZNE), Bonn, Germany

**Keywords:** Neonatal and post-neonatal mortality, Causes of death, Social class, Seasonality, Nineteenth century, Germany

## Abstract

Little is known about social gradients in cause-specific infant mortality in the nineteenth century. To our knowledge, this is the first paper to explore this connection for the time prior to the epidemiologic and demographic transitions. We used the church records of Rostock, an important port city on the Baltic coast in northern Germany, and prepared and merged the baptismal and burial registers of its largest parish (St. Jakobi) for the periods 1815–1836 and 1859–1882. Based on individual-level data (*N* = 16,880), we classified the fathers’ occupations into three social classes and estimated cause-specific infant mortality risks for these groups using event history analysis. We found a clear social gradient in neonatal and post-neonatal mortality. This gradient was driven by waterborne diseases and convulsions, suggesting severe nutritional and sanitation deficits among the lower social classes even before the city began to struggle with worsening living environments following industrialisation and population growth in the second half of the nineteenth century. Our results also suggest that deteriorating environmental conditions affect all parts of the population, leading to an increase of infant mortality rates in all social classes. Improvements in nutritional and sanitary conditions may thus reduce the risk of infant death from infectious diseases.

**Supplementary Information:**

The online version contains supplementary material available at 10.1007/s11111-025-00483-w.

## Introduction

Infant mortality is a widely used indicator of mortality and population health (Gonzalez & Gilleskie, 2017; Masuy-Stroobant & Gourbin, 1995; Reidpath & Allotey, 2003). Its decrease in many industrial countries from the late nineteenth century onwards was one of the principal causes of the increased life expectancy and, hence, of the demographic transition (Chesnais, 1992; Kirk, 1996; Schofield et al., 1991). The determinants of pre-transition, nineteenth-century infant mortality have long been among the key issues of historical demographic research. This special research interest is partly based on the observation that contemporary populations in a number of less developed countries currently face poor sanitary conditions similar to those in Western countries at the dawn of the epidemiologic and demographic transitions (Pozzi & Ramiro Fariñas, 2015). The cause-of-death spectrum in infant and overall mortality prior to the epidemiologic and demographic transitions was characterised by infectious diseases and recurring epidemics (Omran, 1971, 1998). Therefore, the latest rise in the spread and mortality related to infectious diseases, particularly since the onset of the Covid-19 pandemic, has triggered renewed interest into this subject (Janssens, 2021; Petersen et al., 2020; Raftakis, 2022).

In the context of historical infant mortality, several studies have explored social inequality, while others have examined differences by cause of death. However, little is known about the interplay between the two. Given that causes of death may provide deeper insights into the potential reasons for differentials and trends in infant mortality, this is a surprising gap in the research. It may stem from several factors. First, cause-of-death data prior to the twentieth century are generally sparse. Second, the digitisation, transcription and preparation of such data are expensive and time consuming. Third, the sources of data on cause of death frequently do not include useful information on social status. Fourth, many research papers focus on small parishes or short periods (partly due to high transcription costs), resulting in a number of infant death observations that is too small for cause-specific analysis.

Aiming to close this research gap, we used a large, newly-available data source of church records from the Hanseatic city of Rostock, Germany. These data include information on both cause of death and social status for large numbers of residents and describe an urban setting prior to the demographic and epidemiologic transitions when infectious diseases were still ubiquitous, infant mortality was high, and industrialisation and urbanisation were just starting to emerge. After classifying causes of death into four groups and fathers’ occupations into three social classes, we used event history methods to estimate the impact of social class on cause-specific infant mortality.

## Background

### Infant mortality in nineteenth-century Germany

**Fig. 1:**
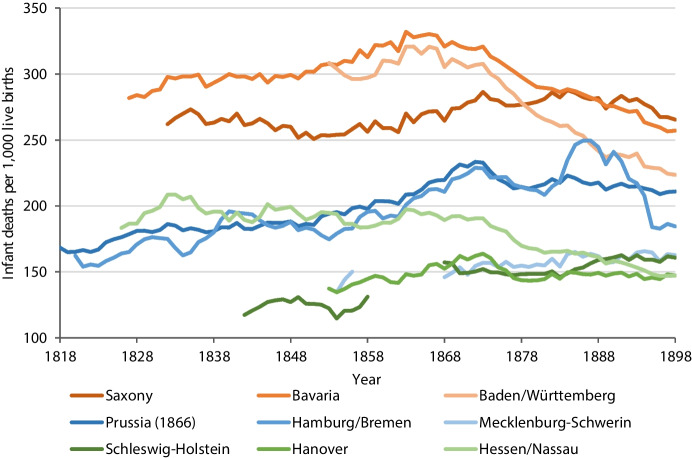
Infant mortality rate in German regions (five-year moving average), 1818–1898. Source: Authors’ calculations based on data provided by Gehrmann (2002, 2011)

The actual beginning of the epidemiologic and demographic transitions in Germany is disputed. While first signs of a transition from the phase of pestilence and famine to a phase of receding pandemics were identified by the 1830s, infants and children were not yet experiencing significant improvements in survival at that time (Spree, 1998). To the contrary, due to diverging trends in infant and child mortality, particularly related to the ongoing spread of infectious diseases, the onset of a sustained increase in life expectancy did not occur before the very late nineteenth century, and even then with considerable regional variation across Germany (Imhof, 1985). There was a northwest-to-southeast divide with the northwest showing the lowest mortality rates (Würzburg, 1887; Würzburg, 1888; Prinzing, 1899, 1900; Imhof, 1981; Gehrmann, 2011). Infant mortality rates in northern German regions like Mecklenburg-Schwerin, Schleswig–Holstein and Hanover were comparable to those in Scandinavia and fluctuated between 100 and 200 per 1,000 live births during the second half of the century (Fig. 1). In parts of southern Germany, by contrast, the rate was more than 300 (i.e. 30% of all live births did not survive the first year of life). Most German regions experienced an increase in infant mortality in the third quarter of the nineteenth century due to worsened living environments as the result of industrialisation, urbanisation and population growth, accompanied by adverse developments in infant care, infant feeding and women’s workload (Gehrmann, 2011). Hessen and Nassau in west-central Germany were the only areas to show an almost continuous (albeit slow) decline in infant mortality from the 1830s onwards, which, like Sweden, began to accelerate in the 1870s (Gehrmann, 2011). The south of Germany (Baden, Württemberg and Bavaria) followed in the 1860s, while infant mortality did not decrease in the north of Germany until the early twentieth century. The decline in infant mortality was a result of improved medical care, public health, sanitation/hygiene, nutrition and work conditions (Imhof, 1981; Vögele & Woelk, 2002).

### The city of Rostock

**Fig. 2:**
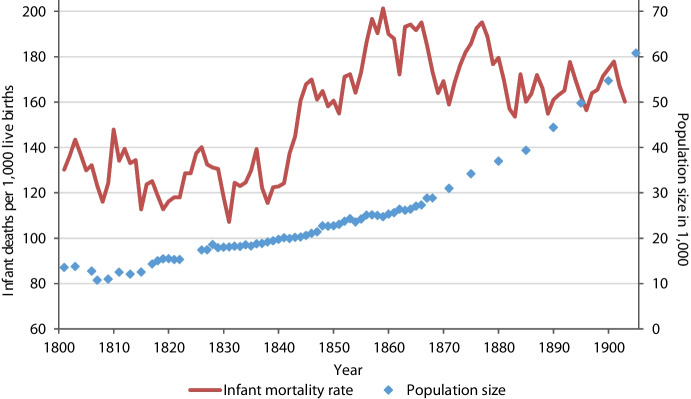
Population size (in thousands) in Rostock (right scale), 1801–1905, and infant mortality rate (five-year moving average) in Rostock (left scale), 1801–1903. Source: Local statistical office of the Hanseatic City of Rostock and Mecklenburg-Schwerin State Calendar for the population size; authors’ calculations for infant mortality based on the baptismal and burial registers of St. Jakobi parish, Rostock

Located at the Baltic coast and shaped by its port and its university, Rostock is the largest and economically most important city in the Mecklenburg region. It was one of the earliest and most influential members of the Hanseatic League (Dollinger, 1970). Like most of northern Germany, the city of Rostock and all of its parishes were Protestant (Evangelical-Lutheran), while Catholics, Jews and other religious groups combined constituted less than 1%, as the censuses of 1819 and 1867 reveal (RAPHIS, 2016). Infant mortality in nineteenth-century Rostock was slightly higher in comparison to the rural areas of Mecklenburg but still lower than in most German regions (Brüning & Balck, 1906; Mühlichen et al., 2015; Paulsen, 1909; Prinzing, 1900; Toch et al., 2011). Infant mortality in Rostock increased significantly between 1841 and 1846 and in the late 1850s (Fig. 2). A peak of 221 infant deaths per 1,000 live births in 1846 (or 170 as a five-year moving average) coincided with a potato blight that destroyed crops over much of Europe. In Mecklenburg, less than half of the usual harvest could be realised (Zadoks, 2008). Failures in potato and grain harvests during 1845–1846, and resulting famines, led to an excess of deaths and pauperisation, especially among landless laborers and rural artisans, who were severely affected by shortages, hunger and death (Zadoks, 2008). These factors contributed to political unrest, the European revolutions of 1848, strong rural–urban migration as well as mass emigration (Haack, 1984; Marschalck, 1973; Zadoks, 2008). Rostock’s population grew continuously with an accelerated growth in the last third of the century. Up to about 1872, Rostock’s population growth was driven primarily by immigration (particularly by young adults from Mecklenburg’s rural subclasses), while natural increase became the leading factor thereafter (Gruber & Scholz, 2018; Szołtysek et al., 2011). The share of people born outside of the city rose from 39.2% in 1819 to 55.9% in 1900. Among people of working age, it even increased to 70% until 1900, while the share remained relatively low among children (Szołtysek et al., 2009, 2011). Rostock’s population growth led to the establishment of new quarters beyond the city walls from the 1850s, which probably contributed to a change in social structures across Rostock’s parishes (Mühlichen & Cilek, 2024). Moreover, in the 1850s, Rostock increasingly struggled with contaminated water, which contributed to the cholera epidemics of 1850 and 1859 (Uffelmann, 1889; Rathaus Rostock, 2017). Infant mortality peaked in 1859 with 249 infant deaths per 1,000 live births (or 201 as a five-year moving average). In reaction to the cholera epidemics, Rostock’s first waterworks was completed in 1867 (Rathaus Rostock, 2017). From this year onwards, infant mortality fluctuated below peak levels, between 150 and 180 per 1,000, interrupted by an increase in the mid-1870s.

### Causes of death

Determinants of infant mortality differ considerably over the course of the first year of life, particularly between the first month and the following eleven months. For this reason, neonatal mortality (considering the first 28 days of life) and post-neonatal mortality (thereafter) are often studied separately. The causes of neonatal mortality are widely related to endogenous factors such as birth weight, foetal age at birth (gestation week), and congenital malformations, whereas post-neonatal mortality is mainly influenced by environmental factors such as nutrition, sanitation/hygiene, socio-economic and climatic conditions (Bourgeois-Pichat, 1951; Breschi et al., 2011; Imhof, 1981; Knodel & Hermalin, 1984; van Poppel et al., 2005).

The historical cause-of-death patterns differed between the neonatal and post-neonatal period as well. Ill-defined causes like ‘weakness’ and ‘wasting’ or congenital disorders and malformations were often recorded in the context of neonatal mortality (Lee & Marschalck, 2002; Reid, 2001; Vögele, 1994). The main risk factor for neonatal death due to weakness is a low birth weight, which is closely related to gestational age and influenced by maternal age at childbirth, poor harvest yields, birth rank, and birth spacing (Imhof, 1981; Kloke, 1997; Knodel, 1988; Knodel & Hermalin, 1984; Würzburg, 1888). In post-neonatal mortality, weakness and wasting were rarely recorded and indicate acute malnutrition (Reid, 2002).

The cause-of-death pattern in post-neonatal mortality was dominated by infectious diseases and epidemics, as was the case for overall mortality prior to the epidemiologic transition (Omran, 1971, 1998; Reid, 2002). Most then-common infections were transmitted either by water and/or food (waterborne diseases) or by air (airborne diseases).

Waterborne diseases include mostly gastro-intestinal infections like cholera, diarrhoea, and gastritis, and were the most frequent cause-of-death group among infants in nineteenth-century Germany (Vögele, 1994). These diseases were closely linked to poor sanitation and inadequate nutrition, and they were particularly prevalent in urbanizing areas (Imhof, 1981; Kintner, 1986; Vögele, 1994; Vögele & Woelk, 2002; Woods et al., 1988). Infant deaths from waterborne diseases were most common in the warm months of the year because breastmilk substitutes were more likely to spoil and water was more likely to be contaminated (Würzburg, 1891; Prausnitz, 1901; Gottstein, 1935; Knodel, 1988: 62; Woods et al., 1988, 1989; Gehrmann, 2002). Whether or not a mother breastfed her child was influenced by her marital and occupational status. Thus, both unmarried mothers and mothers who were extensively involved in their husband’s work (e.g. in an artisan’s shop or on a farm) had less time to care for their children and were more likely to use substitute nutrition (Imhof, 1981; Preston & Haines, 1991: 30; Vögele, 1994). However, breastfeeding habits also varied by region and therefore gave rise to regional differences in infant mortality (Bluhm, 1912; Kintner, 1985; Kloke, 1997; Knodel & Kintner, 1977).

Airborne diseases include respiratory infections such as pneumonia, whooping cough, diphtheria, and measles. As opposed to waterborne diseases, these were most common in the cold months of the year, with poor-quality housing, high housing density, and inadequate clothing being important contributing factors (Derosas, 2009; Lee & Marschalck, 2002; Peiper, 1913; Selter, 1919). The risk of respiratory disease-related early neonatal death was higher for boys than girls due to the lower physical maturity of infant boys’ lungs (Waldron, 1983).

A widespread ill-defined cause of infant death in the nineteenth century is ‘convulsion(s)’. It did not refer to an actual disease but rather to symptoms from a diverse spectrum of diseases, such as gastro-intestinal, respiratory and neurological infections, and metabolic disorders (Vogel & Biedert, 1894; Wienholts et al., 2025). Many scholars have attributed inadequate and unhygienic feeding practices as primary risk factors (Kintner, 1986; Lee & Marschalck, 2002; Prausnitz, 1901; Schlossmann, 1897; Wasserfuhr, 1869), apparently assuming that convulsions were widely related to waterborne infections. However, given the variety of possible underlying diseases, including airborne infections, this conclusion might not be appropriate for every context (Garrett & Reid, 2022; Wienholts et al., 2025). The understanding of convulsions probably varied between the neonatal and post-neonatal period, between places and over time (Walhout, 2010). Therefore, convulsions are usually studied separately.

### Socio-economic differences

One of the most analysed factors in the context of nineteenth-century infant mortality is socio-economic status, which is usually estimated based on the occupation of the child’s father. Although it does not directly affect survival rates, it can influence factors that have a direct effect on infant health, such as breastfeeding habits, the quality of food, parental care, personal hygiene, and sanitary conditions (Bengtsson & van Poppel, 2011; Ekamper & van Poppel, 2019; Gehrmann, 2011; Haines, 1995; Hanssen, 1912; Imhof, 1981; Scott & Duncan, 2000). The effect of socio-economic status is usually expected to be stronger on post-neonatal mortality, as children in the neonatal stage were usually breastfed and thus less exposed to environmental influences (Breschi et al., 2000; Haines, 1985; van Poppel et al., 2005).

The question of whether a social gradient existed in infant mortality has been debated in the existing literature, as has the direction of such a gradient. Some studies have found that socio-economic status had no effect (e.g. Bengtsson, 1999; Knodel, 1988), while others found evidence of better outcomes among the upper social classes (e.g. Derosas, 2003; Molitoris, 2017; Mühlichen et al., 2015; van Poppel et al., 2005; Woods et al., 1988, 1989). In contrast, other studies found that the lower classes benefitted most from the wider practice of breastfeeding in some places (e.g. Fornasin & Rizzi, 2022; Imhof, 1984; Kloke, 1997). These different results confirm that socio-economic status may have significant but variable impacts on infant survival.

To our knowledge, the only studies that broached the interplay of cause-specific infant mortality and social class in the nineteenth century was conducted by Molitoris (2017) for Stockholm, albeit focussing on child mortality (ages 0–9) instead of infant mortality, and Murkens et al. (2022) for Maastricht. However, both works refer to later periods during the demographic and epidemiologic transitions.

To study the impact of the major determinants on infant mortality in a pre-transitional setting, we used church records from Rostock, taking into account 1) both neonatal and post-neonatal mortality, 2) cause-of-death groups according to the main risk factors, and 3) social class as an important environmental variable.

## Data and methods

### Sources

Measuring infant mortality requires data on live births and infant deaths. We used church records from the St. Jakobi parish of Rostock, which are the only transcribed data source that include the necessary information for the city. While the baptismal registers of St. Jakobi include the population at risk (i.e. the live births registered in this parish during the observation period), the burial registers include all infant deaths recorded in the parish. St. Jakobi was by far the largest of the four historical (all-Protestant) parishes of the city and comprised Rostock’s New Town in the west of the city’s historic centre. The proportion of live births in Rostock born in St. Jakobi rose steadily from 39 to 61% between 1827 and 1875 (Mühlichen & Cilek, 2024). While the other three parishes were either wealthy (St. Marien) or poor (St. Nikolai and St. Petri), St. Jakobi was the most balanced in terms of social structure (Mühlichen & Cilek, 2024; Szołtysek et al., 2011). Therefore, it is a reasonable representation of the city.

As is the case with most demographic data, the church records do not provide information on whether and how many infants left the parish after birth (and died elsewhere within the first year of life). The infant mortality rate may therefore be underestimated. The church records of Rostock were compiled by the parish pastor (with the help of his deacon). We have found no evidence that these pastors followed certain rules for diagnosing diseases and distinguishing between live and stillbirths, and whether any guidelines that were used changed between the periods studied.^1^ At least, we could not identify any group-specific under-reporting in the registers. It rather seems that the pastors were devoted to gathering information on births and deaths as complete as possible, as even stillbirths and unbaptised infant deaths from all social classes,^2^ and illegitimate children (sometimes including information on the likely or suspected father) were recorded throughout the observation period.

Furthermore, it is unclear whether pastors consulted doctors in some cases, although the buildings of the medical faculty of the University of Rostock were within walking distance of the church. Given the range of diagnoses recorded and the requirements for keeping church records as set out in ecclesiastical statutes of the period (Gesenius, 1839; Millies, 1895, 1896), it is likely that these pastors had some basic medical knowledge. Although the accuracy of recorded causes of death was not optimal, especially in the early nineteenth century, it seems to have improved over time as diagnoses became more specific and varied (Mühlichen & Cilek, 2024). Even after harmonizing all variant forms of spelling, there were still 150 diseases recorded in our data. In only 60 out of 2,689 infant deaths was the cause of death unspecified or unknown. The trend of improving diagnoses was also observed elsewhere in Europe (e.g. Janssens & Riswick, 2023; Pujadas-Mora, 2024).

### Data preparation

We obtained the transcribed baptismal registers from the RAPHIS (2016) database of the Max Planck Institute for Demographic Research (MPIDR), which grants open access to historical individual-level statistics for Rostock. However, only the periods of 1815–23 and 1863–79 are recorded entirely, i.e. without missing years or cases. Aiming to close the gaps in the database, we added the years that were only partially transcribed and transcribed data for several previously missing periods ourselves using the digitised scans of the church records available from the MPIDR. In the end, we generated a data file containing the periods 1815–1836 and 1859–1882 with information on the date of birth, the date of baptism, the child’s full name, the full names of the parents, the father’s occupation, the legitimacy of birth and (in some cases) the birthplace (only noted if it was outside of Rostock).^3^

For the burial registers, we used a data file created by Mühlichen (2020) which covers 1787–1910 and includes all deaths at age 0–1 (*N* = 10,227). This file includes the date of death, the date of burial, the birthplace (only from 1847 onwards), the child’s full name, the cause of death and age upon death (in days, weeks, months or in some cases approximated as ‘1 year’). In the case of legitimate births, the father’s name and occupation were recorded as well. Regarding illegitimate births, the mother’s name was recorded instead.

To combine the infant deaths with the population at risk and to calculate the exact age at death, we merged the baptismal and burial data into one file. Before doing so, we checked and harmonised the data carefully, particularly regarding the spelling of names, the date of birth, date of death, father’s occupation and legitimacy of birth. The steps of the matching and selection procedure are detailed in the supplementary material. After excluding stillbirths, the merged data file includes 16,880 children who were born in the periods 1815–1836 and 1859–1882, of whom 2,689 died in the first year of life (15.9%), including 823 neonatal and 1,866 post-neonatal deaths.^4^

### Outcome variables and covariates

The main outcome variable is infant death, differentiated by the following causes: convulsions, waterborne, airborne, weakness (including wasting) and other diseases. Waterborne infections include mostly gastro-intestinal diseases like cholera and diarrhoea, whereas airborne infections primarily include respiratory diseases, such as bronchitis and pneumonia. Convulsions and weakness were common ill-defined causes that rather described symptoms than the actual cause and could be related to various diseases. The cause-of-death classification was developed to categorise infant deaths according to major risk factors, using historical sources such as Höfler (1899) and historical German lexica that are available through the Zeno.org (2023) online library. It was influenced by recent works from the *Studying the History of Health in Port Cities* (SHiP) network that established the historical cause-of-death coding scheme ICD10h (Garrett & Reid, 2022; Janssens, 2021; Janssens & Riswick, 2023). Our classification is similar to the ICD10h infant categories (Mühlichen & Cilek, 2024), except that we have reduced the number of disease groups and assigned gastro-intestinal diseases to waterborne infections and respiratory diseases to airborne infections because the vast majority of these diseases are most likely to have an infectious origin in the study context. Table S1 in the supplementary material shows the diseases recorded in the data by cause-of-death group and frequency. In addition, we constructed two further outcome variables for neonatal deaths (up to 28 days of age) and post-neonatal deaths (older than 28 days).

The main variable of interest is social class. To measure its impact, we created an occupational coding scheme using the knowledge provided by studies on historical occupational and social structures in the cities of the German Baltic Sea region (Brandenburg & Kroll, 1998; Brandenburg et al., 1991; Lorenzen-Schmidt, 1996; Manke, 2000) and historical German lexica (Zeno.org, 2023). It is a three-digit system with a [–0] for general occupational groups, a [–1] indicating the lowest social status within that group, and [–2], [–3] and [–4] referring to the higher statuses. We assigned the occupations of the fathers to the following three groups of social classes: Social class A includes high-level officials, merchants, doctors, professors and proprietors, and is referred to in the following as ‘high status’. Social class B, ‘medium status’, includes craftsmen, medium-level officials, steersmen, skippers, teachers, grocers and wagoners. Social class C, ‘low status’, includes labourers, seamen, low-level officials, day labourers, porters, apprentices, servants, factory workers, field workers, fishermen, artists and unknown fathers (particularly in the case of illegitimate births). The complete list of occupations, their codes and their allocation to the social classes is given in Table S2 in the supplementary material. Furthermore, we controlled for sex, period (1815–1836 and 1859–1882), season of birth (warm months: May to September; cold months: October to April), multiple (or single) birth and legitimacy of birth.

### Methods

We analysed the risk of dying in the first year of life using event history analysis based on the Cox proportional-hazards model (Cox & Oakes, 1984).^5^ All exits different from that of interest are treated as right-censored. The analysis time was measured by age in days, from birth to infant death. The age of newborns who survived the first year of life (i.e. no infant death was recorded) was set to the censored time of 365 days. In the case of neonatal mortality, the censored time was 29 days. The analysis of post-neonatal mortality excludes neonatal deaths by definition and therefore involved left-truncated data (i.e. observations start at the age of 29 days). For simplicity, we interpreted hazard ratios as relative risks.

## Results

### Descriptive statistics

Our results show that crude death rates were highest among male infants, the 1859–1882 cohorts and the lowest social class (Table 1). This was true for total infant mortality as well as for neonatal and post-neonatal mortality.

**Table 1:** Live births, infant, neonatal and post-neonatal deaths and crude death rates by sex, period of birth, season of birth, multiple birth, legitimacy of birth and social class in Rostock, 1815–1836 and 1859–1882. Note: Crude death rate is estimated here as the proportion of infants who did not survive the first year of life. Source (as for all following tables and figures): Authors’ calculations based on the baptismal and burial registers of St. Jakobi parish, Rostock

Variable	Category	Live	All infants		Neonatal		Post-neonatal	
		births	Deaths	Rate	Deaths	Rate	Deaths	Rate
*Sex*	Male	8,750	1,492	0.171	479	0.055	1013	0.116
	Female	8,130	1,197	0.147	344	0.042	853	0.105
*Period of birth*	1815–1836	4,279	508	0.119	178	0.042	330	0.077
	1859–1882	12,601	2,181	0.173	645	0.051	1,536	0.122
*Season of birth*	May–September	6,708	1,128	0.168	370	0.055	758	0.113
	October–April	10,172	1,561	0.153	453	0.045	1,083	0.106
*Multiple birth*	Singletons	16,492	2,531	0.153	750	0.045	1,781	0.108
	Twins and triplets	388	158	0.407	73	0.188	85	0.219
*Legitimacy of birth*	Legitimate	13,876	2,041	0.147	619	0.045	1,422	0.102
	Illegitimate	3,004	648	0.216	204	0.068	444	0.148
*Social class*	A: High	1,115	128	0.115	38	0.034	90	0.081
	B: Medium	6,406	958	0.150	266	0.042	692	0.108
	C: Low	9,359	1,603	0.171	519	0.055	1,084	0.116
Total		16,880	2,689	0.159	823	0.049	1,866	0.111

**Fig. 3:**
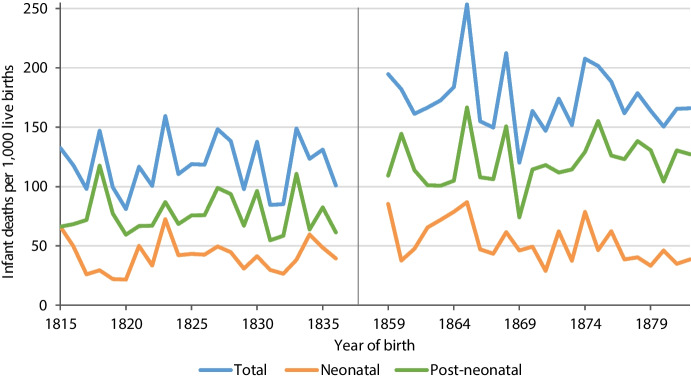
Neonatal, post-neonatal and total infant mortality rates by year of birth in Rostock, 1815–1836 and 1859–1882. Note (as for Fig. 4): Rates are estimated as the proportion of infants born in a year *t* who died within their first year of life (which might occur in years *t* or *t* + *1*)

**Fig. 4:**
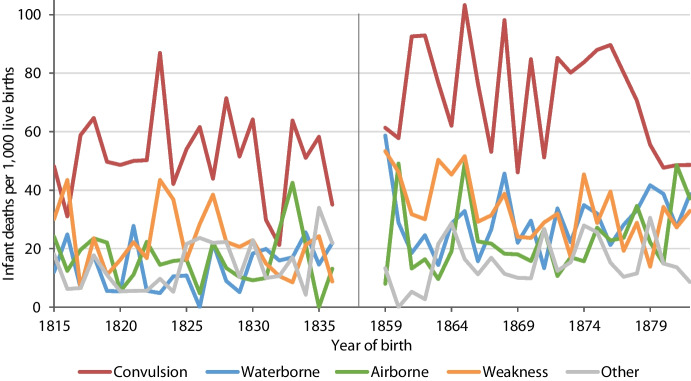
Cause-specific infant mortality rates by year of birth in Rostock, 1815–1836 and 1859–1882

Post-neonatal mortality was higher than neonatal mortality throughout the observation periods, especially between 1859 and 1882 (Fig. 3). The higher level of infant mortality in the later period was strongly associated with an upward shift in post-neonatal mortality. Convulsions were the most common cause of death in almost all birth cohorts, though to a decreasing extent after 1876 (Fig. 4). A total of 1,123 infants died of convulsions, followed by 500 of weakness, 438 of waterborne diseases, 371 of airborne diseases and 257 of other diseases. Infant mortality peaked in 1865, reaching 254 infant deaths per 1,000 live births, mainly due to an increase in convulsions and airborne diseases. Waterborne diseases peaked in 1859 due to a severe cholera epidemic.

**Fig. 5:**
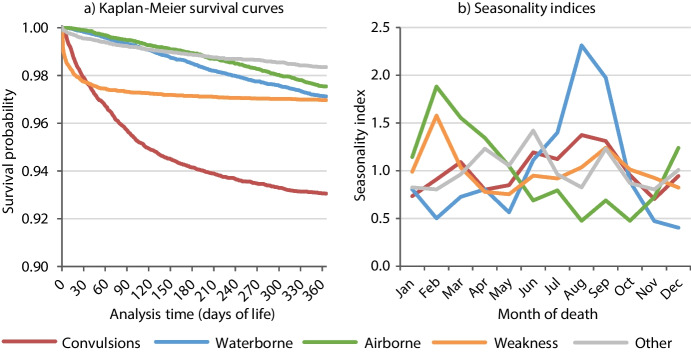
Kaplan–Meier survival curves and seasonality indices by cause-of-death groups in Rostock, 1815–1836 and 1859–1882. Note: Survival curves show the proportion of live births who survived for each day of their first year of life. Seasonality indices are ratios of the monthly numbers of deaths (per day) and the annual numbers of deaths (per day), estimated separately for causal groups

Overall, 84.1% of live births in the 1815–1836 and 1859–1882 periods survived their first year of life, but the level and distribution of survival probabilities by age varied considerably by cause of death (Fig. 5a) and independent variables (Figure S1 in the supplementary material). Mortality from weakness was widely concentrated on the first month of life. For convulsions, mortality was particularly high in the first four months of life. The other survival curves declined more linearly, with waterborne diseases decreasing slightly faster after three months, airborne diseases after six months and other diseases during the first month. In terms of seasonality, waterborne diseases showed a strong summer peak, whereas airborne causes were most common in winter (Fig. 5b). Figure S2 in the supplementary material shows the trends in live births, stillbirths and infant deaths.

### Event history analysis

Since the mortality patterns for the independent variables differed between the first month of life and the ensuing months (Figure S1 in the supplementary material), we applied separate models for the first 28 days of life (neonatal mortality) and the remaining period up to one year (post-neonatal mortality). By doing so, it is possible to focus more specifically on the determinants and causes of infant mortality that differ substantially between the neonatal and post-neonatal stage.

The risk of neonatal death differed by social class, legitimacy, season, period of birth, multiple birth and sex, though not for every cause of death (Table 2a). All-cause mortality was 17% higher among the medium social class (hazard ratio HR = 1.17, *p*-value = 0.365), albeit not statistically significant, and 36% higher (marginally significant) among the low social class (HR = 1.36, *p* = 0.073) as compared to the high social class. Children of single mothers had a 40% higher risk than children of married parents (HR = 1.40, *p* = 0.000). In the birth season from October to April, mortality was 20% lower than between May and September (HR = 0.80, *p* = 0.001). It was 350% higher among multiple births than among single births (HR = 4.50, *p* = 0.000), and 21% higher in 1859–1882 (HR = 1.21, *p* = 0.001) than in 1815–1836. Furthermore, female infants had a 23% lower risk of neonatal death (HR = 0.77, *p* = 0.000) than male ones. The social differences were mainly attributable to convulsions with HR = 2.24 (*p* = 0.028) for the medium and HR = 3.21 (*p* = 0.001) for the low social classes. This social gradient was not evident in other cause-of-death groups. The disadvantage of illegitimate births was highest in the context of airborne (HR = 8.40, *p* = 0.007) and waterborne diseases (HR = 5.57, *p* = 0.001). However, the number of neonatal deaths from waterborne and airborne diseases was very low (25 and 16, respectively) and are thus to interpret with caution. The risk of neonatal death among multiple births was highest for weakness/wasting (HR = 8.81, *p* = 0.000). The seasonal gradient was strongest in waterborne diseases (HR = 0.30, *p* = 0.006) and convulsions (HR = 0.77, *p* = 0.020). The sex gap was also most pronounced in waterborne diseases (HR = 0.26, *p* = 0.008) and convulsions (HR = 0.75, *p* = 0.010). The period effect was only statistically significant in weakness/wasting (HR = 1.41, *p* = 0.008).

**Table 2:**
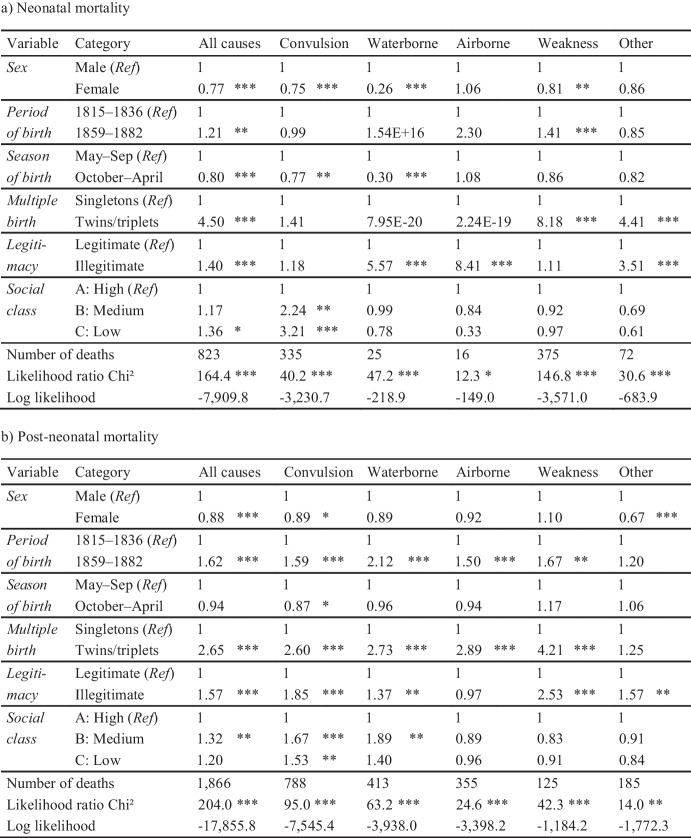
Hazard ratios for neonatal (a) and post-neonatal mortality (b) from Cox proportional-hazards models in Rostock, 1815–1836 and 1859–1882. Note: * *p* ≤ 0.1; ** *p* ≤ 0.05; *** *p* ≤ 0.01. *Ref* indicates the reference category. Number of total observations is 16,880 for neonatal and 16,057 for post-neonatal mortality (without 823 neonatal deaths), degrees of freedom is 7

The risk of post-neonatal death differed by social class, legitimacy, period of birth, multiple birth and sex (Table 2b). Compared to neonatal mortality, the gradients between female and male infants, between the warm and cold birth season, between single and multiple births and between the highest and lowest social class were smaller. However, the shift from the earlier to the later period and the gap between children of married and unmarried parents were stronger. Overall, the risk was 32% (HR = 1.32, *p* = 0.014) higher among the medium social class and 20% (HR = 1.20, *p* = 0.108) higher among the low social class as compared to the high social class. Children of single mothers had a 57% higher risk than children of married parents (HR = 1.57, *p* = 0.000). Multiple births had a 165% higher risk than single births (HR = 2.65, *p* = 0.000). In the birth season from October to April, it was 6% lower than between May and September, though not statistically significant (HR = 0.94, p = 0.180). The risk increased over time by 62% (HR = 1.62, *p* = 0.000) from period 1815–1836 to period 1859–1882. Female infants had a 12% lower risk of post-neonatal death than male infants (HR = 0.88, *p* = 0.008). The social differences in post-neonatal mortality were mainly attributable to waterborne diseases (with HR = 1.89, *p* = 0.015 for the medium and HR = 1.40, *p* = 0.202 for the low social class) and convulsions (with HR = 1.67, *p* = 0.009 for the medium and HR = 1.52, *p* = 0.031 for the low social class). The sex gradient was strongest among other causes (HR = 0.67, *p* = 0.008). The period effect was evident in all cause-specific groups and particularly strong for waterborne diseases (HR = 2.12, *p* = 0.000), albeit not statistically significant for other causes (HR = 1.20, *p* = 0.294). The season of birth had little impact on post-neonatal mortality and was only statistically significant in the context of convulsions (HR = 0.87, *p* = 0.061). The increased risk of post-neonatal death for multiple births was particularly pronounced for weakness/wasting (HR = 4.21, *p* = 0.000). The effect of legitimacy was also strongest for weakness/wasting (HR = 2.53, *p* = 0.000).

To examine the interaction between period and social class, Table 3 shows the hazard ratios 1) for the period 1815–1836, 2) for the change in the high social class between the first and the second period 1859–1882, and 3) how the medium and low social classes differed from the high social class regarding this change from the first to the second period. The change in the high social class between the two periods in the second part of the table is used as the reference category for the third part of the table to show the relative deviation from this class in the other two classes. The results show that the social classes did not differ significantly in the change of infant mortality between the periods 1815–1836 and 1859–1882, either in (a) neonatal or (b) post-neonatal mortality. However, there was a tendency towards a lower increase in neonatal mortality in the medium (HR = 0.69, *p* = 0.371) and low classes (HR = 0.68, *p* = 0.336) than in the highest social class, which was especially driven by trends in weakness/wasting and other causes.

**Table 3:**
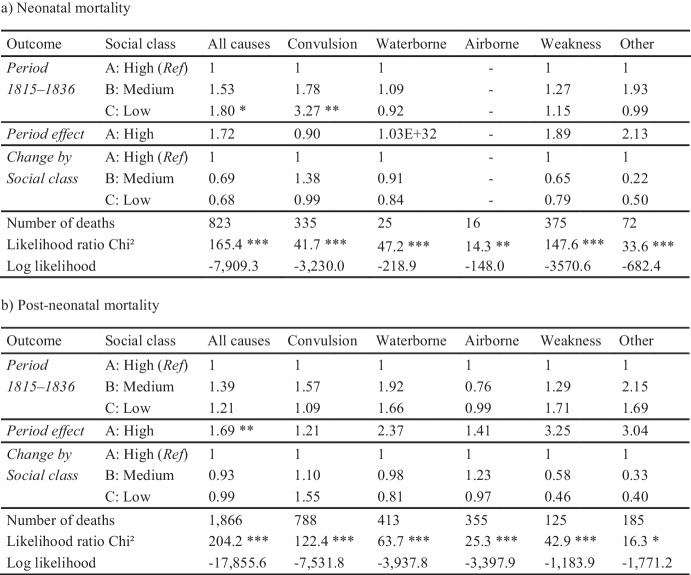
Interaction in neonatal (a) and post-neonatal mortality (b) between period and social class: Change between the periods 1815–1836 and 1859–1882 by social class in Rostock, Hazard ratios from Cox proportional-hazards models. Note: * *p* ≤ 0.1; ** *p* ≤ 0.05; *** *p* ≤ 0.01. *Ref* indicates the reference category. The numbers of airborne diseases in neonatal mortality were too small for analysis. Sex, season of birth, multiple births and legitimacy of birth were included as control variables. Number of total observations is 16,880 for neonatal and 16,057 for post-neonatal mortality, degrees of freedom is 9

## Discussion

Based on church records from Rostock, Germany, we found a strong social gradient in both neonatal and post-neonatal infant mortality. This social gradient was particularly strong in deaths due to convulsions and—in post-neonatal mortality only—to waterborne diseases. Maternal marital status (legitimacy of birth) is an important intervening variable. It attenuates the neonatal and post-neonatal mortality risks of lower-class infants. The social gradient did not vary significantly between the first period (1815–1836) and the second (1859–1882).

### Social gradients

Our data confirmed the existence of a social gradient, and found that it was more pronounced in neonatal than in post-neonatal deaths. This result confirms the finding by Mühlichen et al. (2015) for early-nineteenth-century Rostock (1815–1829). Overall, the infant mortality risk was higher for the lower social classes than for the highest one and higher among infants born out of wedlock than among legitimate births. Including illegitimate births in the lowest social class did not bias the results, as legitimacy was included as a separate variable and thus controlled for in the model. Without its inclusion, the social gradient would appear even stronger, as indicated in Figure S1 (supplementary material). The social gradient was further attenuated slightly by the inclusion of the multiple birth variable, as the risk of infant death among twins and triplets was higher for the two lower social classes.

Social differences in infant mortality were also found in several other places in Europe but mostly in closer connection with post-neonatal mortality (e.g. Breschi et al., 2000; Haines, 1985; van Poppel et al., 2005). Nonetheless, some studies also found significant social differences in neonatal mortality, albeit to varying directions (Derosas, 2003, 2009; Fornasin & Rizzi, 2022; Landers, 1993).

In neonatal mortality, the social gradient was driven by convulsions. In post-neonatal mortality, it was attributable to both, convulsions and waterborne diseases, whereas social class had no significant effect in the other disease groups. Our results confirm previous research about nutrition and sanitation as principal determinants for infant mortality, since these two factors were strongly connected with waterborne diseases and – to a disputed degree – with convulsions (Kintner, 1986; Lee & Marschalck, 2002; Preston & Haines, 1991; Vögele, 1994). With respect to nutrition, breastfeeding is a protective factor for infant survival because it helps to maintain maternal passive immunity for longer and prolong birth intervals (Bengtsson, 1999; Scott & Duncan, 2002, pp. 147–157). In contrast, artificially fed infants lose this natural protection sooner and are more likely to come into contact with contaminated food. Therefore, differences in breastfeeding practices are seen as one of the major determinants of nineteenth-century infant mortality and its regional variation (Kintner, 1985; Kloke, 1997; Knodel & Kintner, 1977). With respect to sanitation, geographic variations in the supply of clean water were found to be important influencing factors of infant mortality differentials as well (Jaadla & Puur, 2016; Kesztenbaum & Rosenthal, 2017; van Poppel & van der Heijden, 1997).

Even though Uffelmann (1889) reported that inappropriate food was rarely used in Rostock at that time in favour of breastfeeding, our results indicate that the nutritional and/or sanitary conditions varied noticeably among social groups in nineteenth-century Rostock, as has also been argued with regard to other places in Europe (e.g. Haines, 1995; Kloke, 1997; Oris et al., 2004; Scott & Duncan, 2000). Regarding sanitation, the access to clean water probably varied in the neighbourhoods of nineteenth-century Rostock, which presumably also differed in their social structure (as has been shown elsewhere in Europe at this time) (Ekamper & van Poppel, 2019). Thus, residential segregation in connection with water quality and sanitary conditions could be another explanation for the higher mortality from waterborne diseases and convulsions among infants in Rostock’s lower social classes.

Our results suggest that infants of lower social status were probably more exposed to breastmilk substitutes, malnourishment and unsafe water. While the vast majority of deaths related to waterborne diseases (as well as airborne and ‘other’ diseases) took place in the post-neonatal stage, convulsions were also relatively frequent as cause of death in neonatal mortality. The social gradient in convulsions was even stronger in neonatal mortality than in post-neonatal mortality, which suggests unhygienic conditions surrounding childbirth and/or inappropriate feeding among the lower social classes (Lee & Marschalck, 2002; Prausnitz, 1901; Schlossmann, 1897; Wasserfuhr, 1869). Since waterborne and airborne infections should be relatively rare in the neonatal phase due to maternal passive immunity, the high proportion of convulsions in neonatal deaths might be an indication of neonatal tetanus, which was usually caused by an infection of the umbilical stump and led to death within two weeks after birth (Roper et al., 2007; Vogel & Biedert, 1894).

Our results also point to the importance of the child’s legitimacy status in explaining social differences. Unmarried mothers who had to earn their own living and were more at risk of suffering from poverty were found to be more likely to use poor artificial nutrition instead of breastfeeding (Imhof, 1981; Preston & Haines, 1991, p. 30; Vögele, 1994), which could explain the high excess mortality from waterborne diseases and post-neonatal weakness. The high excess mortality from airborne diseases might be related to their living environment in dense quarters.

### Changes between the periods

As shown by Mühlichen et al. (2015), infant mortality increased considerably in Rostock in the 1840s and 1850s, which is exactly the time between our two study periods. The second period marks the beginning of urbanisation and accelerated population growth. The results of the interaction model show that the increase in infant mortality was similar for all social classes. The results also show that the deficits in nutrition, sanitation and maternal care in the low social class were evident even before industrialisation (coupled with population growth) had led to worsening living environments. This deterioration then affected all parts of the population.

Even though not statistically significant, we found that the increase in neonatal mortality was more pronounced in the highest social class. There are various potential reasons for this: On the one hand, in many places in nineteenth-century Germany, the upper social class was more likely to use substitute nutrition instead of breastfeeding their infants (Imhof, 1984). It is possible, that such a trend evolved in Rostock in the middle of the nineteenth century as well. On the other hand, residential patterns with respect to sanitary conditions also had a considerable impact (Ekamper & van Poppel, 2019). The residential patterns according to social classes have not been analysed for Rostock yet, but Szołtysek et al., (2009, 2011) found that Rostock’s population growth at that time was driven by immigration of young low-status workers from the rural hinterland. As a result, Rostock expanded beyond the city walls with the establishment of two new quarters in the 1850s. *Steintor-Vorstadt*, a mansion district close to Rostock’s Old and Middle Town areas, particularly attract ed Rostock’s upper class, while *Kröpeliner-Tor-Vorstadt*, which is close to Rostock’s New Town (St. Jakobi), was intended for the working class (Mühlichen & Cilek, 2024). These developments led to a change in social structure in Rostock and St. Jakobi in particular. Although all social classes increased in absolute numbers, the share of the high and medium social classes among the observed infants decreased in St. Jakobi from the first to the second period (from 9 to 6% for the high class and from 41 to 37% for the medium class), while the share of the low class increased from 50 to 57%, as additional analyses of the data reveal. Therefore, it is likely that the upward shift of infant mortality is partly related to a change in social structure, which also affected the upper class that came increasingly into contact with low-status groups and their associated diseases, which possibly also affected infants, especially when they were not breastfed.

### Other associations

Multiple births have been shown to be associated with an increased risk of infant mortality, particularly neonatal mortality, because of the greater likelihood of prematurity and the strain on resources (Scalone & Samoggia, 2018; Torres et al., 2023). This is reflected in our results, which show that the highest risk of death among multiple births is associated with weakness in the first four weeks of life.

Infants born between October and April had a significantly lower risk of death than those born in the warmer season. This applies to both the neonatal and post-neonatal periods and confirms the results of an earlier study for Denmark (Doblhammer & Vaupel, 2001). On the one hand, mothers who gave birth in the fall and early winter had access to rich foods and fresh fruits and vegetables during most of their pregnancy; those who gave birth in the spring and early summer experienced longer periods of inadequate nutrition. A study for Vienna, Austria, between 1865 and 1930 showed that infants born between September and November had significantly higher birth weights than those born in the other months of the year (Ward, 1987). Infants born during the cold season also had a lower risk of suffering from gastro-intestinal diseases due to spoiled food, especially if they were not breastfed. Our results support these explanations with a particularly strong effect on neonatal mortality for waterborne diseases and convulsions, as well as for convulsions in the post-neonatal period. On the other hand, infants born during the cold season had a higher risk of developing respiratory infections at a very early age, which is reflected in their increased risk of dying from airborne diseases during the neonatal period in our study. In other places, this disadvantage led to higher neonatal mortality among infants who were born in winter (Fornasin & Rizzi, 2022; Karlsson et al., 2021; Scalone & Samoggia, 2018).

Neonatal and post-neonatal mortality were significantly lower for females than for males, which is a typical result for European cities in the nineteenth century. This female advantage might be due primarily to genetic factors. X-linked immunoregulatory genes appear to contribute to greater resistance to infectious diseases for females (Waldron, 1983).

### Limitations

Despite the contribution of our findings, the study is characterized by several limitations. The first potential bias relates to the quality of the source material, particularly with regard to the accuracy of recorded diseases, occupations and the distinction between live and stillbirths (see *Sources*). Second, we cannot determine whether and how many infants left the parish and died elsewhere in the first year of life and whether this was different across social groups. Mühlichen et al. (2015) found a lower infant mortality risk for illegitimate new-borns compared to legitimate ones in St. Jakobi in the years 1815–29. This was not statistically significant, though. As a possible explanation, they argued that unmarried mothers were more likely than married mothers to move to their parents in the countryside, or hand their children over to them or to other intimates or institutions. In such cases, infant deaths were usually not recorded in St. Jakobi. In our study periods, the mortality risk is significantly higher among illegitimate than legitimate infants. The slight survival advantage of illegitimate newborns in 1815–1829 is reversed by adding the years 1830–36 alone. Nonetheless, the risk of infant death among certain social groups might be underestimated.

Third, our results for the social gradient are shaped by our occupational classification. While we appreciate previous efforts to classify historical occupations, such as HISCO/HISCLASS (van Leeuwen & Maas, 2011; van Leeuwen et al., 2002), we decided to create an own coding scheme that is less complex, easier comprehensible and matches the social hierarchy of Rostock better. According to Manke (2000, pp. 369–371), Rostock’s political and economic elite consisted of merchants, brewers and lawyers, whereas workmen and day labourers formed the lower class, and craftsmen were very heterogeneous in their social structure. We considered this in our classification but some uncertainties remain. For example, we included all merchants (‘Kaufmann’) in the highest social class although some may have been relatively poor. In addition, we put all master craftsmen, journeymen and craftsmen of unspecified rank into the medium social class because the differentiation was not accurate enough in the registers (Table S2 in the supplementary material). A comparison of our classification with HISCLASS-5 is shown in Table S3 (supplementary material).

Fourth, the results can generally be influenced by the selection of the model. However, we performed a sensitivity analysis using competing-risks regression according to Cleves et al. (2010, pp. 365–391), which did not alter our results compared to the Cox models.

## Conclusion

Using newly-available data from the city of Rostock that feature sufficient numbers of cases as well as information on cause of death, age upon death and father’s occupation, it was possible to classify cause-of-death groups and social classes and to run separate models for neonatal and post-neonatal mortality. To our knowledge, this is the first study to examine the association between social class and cause-specific infant mortality prior to the epidemiologic and demographic transitions. These rare insights may be applicable to less developed countries currently facing poor sanitation, accelerated population growth and rapid urbanisation. Aside from targeted medical interventions, improvements in nutritional and sanitary conditions can reduce the risk of infant death from infectious diseases, such as by raising breastfeeding rates and access to clean water (Kotloff et al., 2018). All social groups benefit from these improvements. However, the reverse is also true, and deteriorating environmental conditions may affect all parts of the population, thus raising infant mortality rates in all social classes. Malnutrition and poor access to clean water may still be the main causes of excess infant mortality in less developed countries (Boschi-Pinto et al., 2008; Kotloff et al., 2018).

## Supplementary Information

Below is the link to the electronic supplementary material.

Supplementary file1 (PDF 1.03 MB)

## Data Availability

The transcribed baptismal and burial records of St. Jakobi parish, Rostock, are available in the RAPHIS (2016) database of the Max Planck Institute for Demographic Research (MPIDR). Scans of the original records are also available at MPIDR. The extended harmonised time series and the merged data file used for this study can be obtained from the corresponding author upon reasonable request.

## Footnotes

1 Figure S2 in the supplementary material does not suggest a sudden definitional shift in either live births or stillbirths.

2 Stillbirths and unbaptized infant deaths were recorded consistently in the burial registers. Until November 1876, they were also included in the baptismal registers.

3 Maternal age was mostly not reported in the sources.

4 We have kept four infant deaths in the final data file that were recorded as stillbirths in the burial registers but turned out to be early neonatal deaths after identifying the same cases in the baptismal registers.

5 All data entries, error corrections and the harmonisation of the baptismal and burial data were conducted using Excel. We used SAS and R for the matching procedures and SPSS to process the data for the survival analysis. The survival analysis itself was performed using Stata.
