# Supplementary material for: Social differences in cause-specific infant mortality at the dawn of the demographic transition: New insights from German church records
Source: Popul Environ. 2025 Mar 13;47(1):15. doi: 10.1007/s11111-025-00483-w (PMC11906567; doi:10.1007/s11111-025-00483-w)
Supplement: Supplementary file 1 — Supplementary file1 (PDF 1.03 MB) [file 11111_2025_483_MOESM1_ESM.pdf]

Social differences in cause-specific infant mortality at the dawn of the demographic transition: New insights from German church records

Michael Mühlichen · Gabriele Doblhammer

Supplementary material

Data matching and selection

To conduct this study, the baptismal and burial registers of Rostock's St. Jakobi parish had to be merged. This was necessary for two reasons: First, we needed the population at risk included in the baptismal data and the information about infant deaths included in the burial data for survival analysis. Second, we needed the date of birth from the baptismal data and the date of death from the burial data to calculate the exact span of days from birth to death, since the given age upon death was often not accurate enough to separate infant deaths from non-infant deaths and neonatal deaths from post-neonatal deaths.

In preparation for the matching process, we limited the baptismal data to the 1815–1836 and 1859–1882 periods, and the burial data to the 1815–1837 and 1859–1883 periods. In addition, we harmonised the spelling variants of the surnames and first names to increase the chance of successful matches.

We applied three different matching procedures to reach a maximum linkage quota. First, we conducted a matching procedure in SAS that linked persons from both files if the first two letters of their surname, the first two letters of their first name and their sex were identical. Second, we conducted a fuzzy matching in R that linked persons with an almost identical surname and first name to identify those matches with differences in the first or second letters of their names, or incorrect sex. Third, we conducted another matching procedure in SAS that merged persons if the first four letters of their surname as well as their sex were identical. The aim of this was to find identical persons even if the order of their first names was recorded differently in the registries.

In all three procedures, the year of birth had to be approximately the same (+/-2). The aggregated list of matches included many individuals who were matched more than once. Therefore, we checked this list carefully and deleted all incorrect matches. In addition, we checked the lists of unmatched infant deaths and unmatched live births manually to find identical individuals with a difference in their surname, e.g. if the mother's surname was recorded as the child's surname instead of the father's in one of the two registries.

After excluding stillbirths and children born outside of Rostock, 90.2% of all infant deaths recorded in the burial registers were successfully linked to a live birth in the baptismal register. The unmatched infant deaths included 113 children who died before their baptism. Since children who died before their baptism were no longer recorded in the baptismal registers from 1 December

1876, we added them manually to the merged data file from the burial registers in order to avoid under-coverage of the population at risk in the last years of the study period. The age upon death in these cases was mostly given in days or weeks, being accurate enough for our analyses even without further information from baptismal records. 174 cases in the burial registers could not be linked to the baptismal registers. We did not include these cases as we assume that they were likely born in another place. We also excluded Catholics (6 cases) as they were baptised elsewhere (usually in Schwerin) and we do not know whether infant deaths from this religious minority were systematically recorded in Rostock or possibly elsewhere, too. Moreover, we do not know how many infants migrated from Rostock and died in another place, and were thus not recorded in Rostock's burial registers, which leaves room for under-estimation of infant mortality. Under-estimation of infant deaths could theoretically also appear when burials of infants born in one parish were registered in another one. However, in Rostock, burials were recorded in accordance with the parish they belonged to, not according to the burial ground. The two large burial grounds at this time ('Alter Friedhof' and 'St. Gertrud') were open for people from all Rostock parishes.

After calculating the age upon death from the date of birth and the date of death, 72 cases turned out to be one year or older at death, which we recoded as 'no infant death'. Moreover, we excluded children who were one year and older at baptism from the merged data file, as it is highly probable that they were not born in Rostock and thus do not belong to the population at risk. All in all, the merged data file includes 16,880 live births, of which 2,689 died in the first year of life (15.9%).

Cause-of-death classification

Table S1 Cause of death by disease group among infants born in Rostock, St. Jakobi parish, 1815–1836 and 1859–1882.

Cause of death		Disease group				
German (harmonised)	English translation	Convulsion	Waterborne	Airborne	Weakness	Other
[Keine Angabe]	[Blank]	.	.	.	.	58
Augenübel	Eye disorder	.	.	.	.	1
Ausschlag	Rash	.	.	.	.	1
Auszehrung	Wasting	.	.	.	27	.
Backenkrampf	Cheek convulsion	.	.	.	.	2
Bald nach der Geburt gestorben	Died soon after birth	.	.	.	2	.
Blasenausschlag	Blistering eruption	.	.	.	.	1
Blattern	Smallpox	.	.	4	.	.
Bleichsucht	Chlorosis	.	.	.	1	.
Blutarmut	Anaemia	.	.	.	4	.
Blutverlust	Blood loss	.	.	.	.	1
Bräune	Diphtheria	.	.	9	.	.
Brechdurchfall	Cholera	.	18	.	.	.
Brechleiden	Nausea	.	1	.	.	.
Brechruhr	Cholera	.	6	.	.	.
Bronchialeiterung	Bronchial suppuration	.	.	1	.	.
Bronchialkatarrh	Bronchial catarrh	.	.	1	.	.
Bruch	Hernia	.	1	.	.	.
Brustbräune	Angina pectoris	.	.	2	.	.
Brustentzündung	Chest inflammation	.	.	7	.	.
Brustfieber	Chest fever	.	.	1	.	.
Brustkatarrh	Chest catarrh	.	.	2	.	.
Brustkrampf	Chest convulsion	.	.	29	.	.
Brustkrankheit	Chest disease	.	.	41	.	.
Brustleiden	Chest disorder	.	.	11	.	.
Brustübel	Chest disorder	.	.	9	.	.
Brustverschleimung	Chest catarrh	.	.	8	.	.
Cholera	Cholera	.	17	.	.	.
Cholerine	Cholera	.	5	.	.	.
Darmkatarrh	Intestinal catarrh	.	1	.	.	.
Diarrhoe	Diarrhoea	.	4	.	.	.
Diphtherie	Diphtheria	.	.	2	.	.
Drüsen	Glands	.	.	.	.	13
Drüsenkrankheit	Glandular disease	.	.	.	.	13
Drüsenleiden	Glandular disorder	.	.	.	.	2
Durch die Amme erdrückt	Crushed by the wet nurse	.	.	.	.	1
Durch Operation bei der Entbindung	Due to surgery during delivery	.	.	.	.	1
Durchfall	Diarrhoea	.	27	.	.	.
Eiterung	Suppuration	.	.	.	.	1
Englische Krankheit	Rickets	.	.	.	4	.
Entkräftung	Inanition	.	.	.	4	.
Entzündung	Inflammation	.	.	.	.	4
Entzündung des Zellgewebes	Inflammation of the hip joint	.	.	.	.	1
Erkrankung des Hüftgelenks	Inflammation of cellular tissue	.	.	.	.	1
Fieber	Fever	.	.	.	.	1
Folge der Masern	Effect of measles	.	.	1	.	.

Frieseln	Strophulus	.	.	.	.	11
Frühgeburt	Premature birth	.	.	.	2	.
Gehirnentzündung	Cerebral inflammation	.	.	.	.	21
Gehirnkrampf	Cerebral convulsion	.	.	.	.	2
Gehirnleiden	Brain disorder	.	.	.	.	3
Gehirnschlag	Stroke	.	.	.	.	1
Gelbsucht	Jaundice	.	.	.	.	3
Geschwür	Ulcer	.	.	.	.	2
Gleich nach der Geburt gestorben	Died right after birth	.	.	.	13	.
Grippe	Influenza	.	.	1	.	.
Halsbräune	Diphtheria	.	.	9	.	.
Halsentzündung	Pharyngitis	.	.	2	.	.
Halsgeschwulst	Throat ulcer	.	.	1	.	.
Halsleiden	Throat disorder	.	.	1	.	.
Hautausschlag	Rash	.	.	.	.	2
Hautentzündung	Skin inflammation	.	.	.	.	2
Hautkrankheit	Skin disease	.	.	.	.	2
Herzentzündung	Heart inflammation	.	.	.	.	1
Herzleiden	Heart disorder	.	.	.	.	5
Herzschlag	Heart apoplexy	.	.	.	.	2
Hirnhautentzündung	Meningitis	.	.	.	.	2
Husten	Cough	.	.	10	.	.
Husten und Zufall	Cough and accident	.	.	1	.	.
Im Bett erdrückt	Crushed in bed	.	.	.	.	1
Innere Geschwüre	Internal ulcers	.	.	.	.	1
Katarrh	Catarrh	.	.	2	.	.
Kein Anus	Absence of anus	.	.	.	.	1
Kein Hirnschädel	Absence of vault of skull	.	.	.	.	1
Keine Backen im Mund, sehr kurze Zunge	Absence of cheeks in mouth, very short tongue	.	.	.	.	1
Keuchhusten	Whooping cough	.	.	56	.	.
Keuchhusten und Krampf	Whooping cough and convulsion	.	.	1	.	.
Kindertyphus	Children's typhoid fever	.	1	.	.	.
Knochenfraktur	Bone fracture	.	.	.	.	1
Kopfkrampf	Head convulsion	.	.	.	.	3
Kopfkrampf und Zahnen	Head convulsion and teething	.	.	.	.	1
Kopfkrankheit	Head disease	.	.	.	.	15
Kopfschaden	Head deformity	.	.	.	.	1
Kopfschlag	Stroke	.	.	.	.	1
Krampf/Krämpfe	Convulsion(s)	1,123	.	.	.	.
Krampf und Zahnen	Convulsion and teething	.	1	.	.	.
Krampfschlag	Cerebral convulsion	.	.	.	.	2
Leberleiden	Liver disease	.	1	.	.	.
Luftröhrenentzündung	Tracheal inflammation	.	.	1	.	.
Lungenentzündung	Pneumonia	.	.	82	.	.
Lungenkatarrh	Lung catarrh	.	.	11	.	.
Lungenkrampf	Lung convulsion	.	.	1	.	.
Lungenkrankheit	Lung disease	.	.	1	.	.
Lungenleiden	Lung disorder	.	.	6	.	.
Lungenschlag	Pulmonary embolism	.	.	4	.	.
Lungenübel	Lung disorder	.	.	8	.	.
Lungenverschleimung	Lung inflammation	.	.	1	.	.
Magendarmentzündung	Gastroenteritis	.	1	.	.	.
Magenentzündung	Gastritis	.	19	.	.	.

Magenfieber	Gastric fever	.	1	.	.	.
Magengeschwulst	Gastric ulcer	.	1	.	.	.
Magenkatarrh	Gastric catarrh	.	6	.	.	.
Magenkrampf	Gastric convulsion	.	24	.	.	.
Magenkrankheit	Gastric disease	.	3	.	.	.
Magenleiden	Gastric disorder	.	33	.	.	.
Magenschwäche	Gastric insufficiency	.	2	.	.	.
Magenübel	Gastric disorder	.	51	.	.	.
Magenverschleimung	Gastric inflammation	.	1	.	.	.
Masern	Measles	.	.	23	.	.
Mundfäule	Stomatitis	.	.	2	.	.
Mundkrampf	Lockjaw	.	.	.	.	1
Nabelbruch	Umbilical hernia	.	1	.	.	.
Nabelentzündung	Omphalitis	.	.	.	.	2
Nierenleiden	Kidney disorder	.	.	.	.	1
Operation	Surgery	.	.	.	.	1
Rachenbräune	Diphtheria	.	.	13	.	.
Rippenfellentzündung	Pleurisy	.	.	1	.	.
Rose	Erysipelas	.	.	.	.	7
Rotlauf	Erysipelas	.	.	.	.	1
Rückenleiden	Spinal disorder	.	.	.	.	1
Rückenmarksleiden	Spinal cord disorder	.	.	.	.	2
Ruhr	Dysentery	.	11	.	.	.
Scharlach	Scarlet fever	.	.	.	.	7
Scharlachfrieseln	Scarlet strophulus	.	.	.	.	3
Schlag	Stroke	.	.	.	.	9
Schlag und Zahnen	Stroke and teething	.	.	.	.	1
Schlagfluss	Stroke	.	.	.	.	4
Schleimfieber	Typhoid fever	.	2	.	.	.
Schwäche	Weakness	.	.	.	439	.
Schwamm	Sponge	.	.	.	.	16
Schwamm und Brustkrankheit	Sponge and chest disease	.	.	.	.	1
Schwindsucht	Consumption	.	.	4	.	.
Syphilis	Syphilis	.	.	.	.	5
Totgeburt	Stillbirth	.	.	.	4	.
Typhus	Typhoid fever	.	1	.	.	.
Unbekannt	Unknown	.	.	.	.	2
Unterleibsentzündung	Inflammation of lower body organs	.	1	.	.	.
Unterleibsleiden	Disorder of lower body organs	.	1	.	.	.
Venerisches Gift der Amme	Venereal poison of the wet nurse	.	1	.	.	.
Verblutung und Mord	Exsanguination and murder	.	.	.	.	1
Wassersucht	Hydropsy	.	1	.	.	.
Wurmfieber	Enteritis	.	1	.	.	.
Zahnen	Teething	.	84	.	.	.
Zahnen und Krampf	Teething and convulsion	.	2	.	.	.
Zahnfieber	Dental fever	.	1	.	.	.
Zahnhusten	Dental cough	.	.	1	.	.
Zahnkrampf	Dental convulsion	.	106	.	.	.
Zellgewebeeiterung	Suppuration of cellular tissue	.	.	.	.	1
Zellgewebekrankheit	Disease of cellular tissue	.	.	.	.	1
Total		1,123	438	371	500	257

Occupational classification

Table S2 Father's occupation by social class and frequency among live births in Rostock, St. Jakobi parish, 1815–1836 and 1859–1882.

Group	Code	German description	English translation	Class	N[a]
1) Trade and administration					
Trade	100	Händler[b]	Tradesman[b]	B	59
	101	Handlungsgehilfe, -diener, -reisender	Merchant's apprentice, travelling salesman	C	9
	102	Krämer, Warenhändler	Grocer, shopkeeper	B	165
	103	Kaufmann	Merchant	A	527
Carrying trade	111	Kutscher, Sänftenträger	Cabman, litter bearer	C	24
	112	Fuhrmann	Wagoner	B	189
	113	Fuhrunternehmer, Droschkenbesitzer	Haulage contractor, cab owner	A	5
Gastronomy	121	Kellner, Portier, Büfettier	Waiter, porter, bartender	C	0
	122	Koch, Kantinenpächter	Cook, canteen tenant	C	7
	123	Gastwirt, Herbergierer	Innkeeper	B	160
	124	Hotel- oder Gasthofbesitzer, Hoteldirektor	Hotel or inn owner, hotel director	A	0
Bank, insurance	131	Bank- oder Versicherungsdiener	Bank or insurance servant	C	9
	132	Bank- oder Versicherungsbeamter	Bank or insurance official	B	7
	133	Bank- oder Versicherungsinspektor	Bank or insurance inspector	A	4
	134	Bank- oder Versicherungsdirektor	Bank or insurance director	A	0
Administration, justice	141	Beamter im unteren Dienst	Low-level official	C	66
	142	Beamter im mittleren Dienst	Middle-level official	B	73
	143	Beamter im höheren Dienst	High-level official	A	211
	144	Hochrangiger Beamter	Top-ranking official	A	113
Post	151	Postbeamter im unteren Dienst	Low-level post official	C	29
	152	Postbeamter im mittleren Dienst	Middle-level post official	B	18
	153	Postbeamter im höheren Dienst	High-level post official	A	0
Security	161	Polizei- oder Feuerwehrdiener, Wächter	Police or fire servant, watchman, guard	C	9
	162	Mittlerer Polizei- oder Feuerwehrbeamter	Middle-level police or fire officer	B	6
	163	Höherer Polizei- oder Feuerwehrbeamter	High-level police or fire officer	A	0
Education, science	171	Student, Famulus, Schul- oder Universitätsdiener	Student, famulus, school or university servant	C	2
	172	Lehrer, Dozent, Wissenschaftler (ohne Doktortitel)	Teacher, lecturer, scientist (without doctoral degree)	B	111
	173	Doktor, Professor	Doctor, professor	A	96
	174	Schuldirektor, Universitätsrektor	School or university director	A	3
Medicine, health	181	Krankenwärter, Pflegekraft, Masseur	Nurse, caregiver, masseur	C	17
	182	Chirurg, Zahnarzt, Magnetiseur, Tierarzt	Surgeon, dentist, magnetiser, veterinarian	B	12
	183	Doktor der Medizin	Doctor of medicine	A	31
	184	Hospitalmeister	Hospital administrator	A	5
Church	191	Kirchendiener	Church servant	C	24
	192	Diakon, Kantor, Magister	Deacon, cantor, magister	B	10
	193	Pastor	Pastor	B	2
	194	Kirchenvorsteher	Church leader	A	0
					2,003
2) Transport and military					
Railways	201	Bahnbeamter im unteren Dienst	Low-level railway official	C	28
	202	Bahnbeamter im mittleren Dienst	Middle-level railway official	B	7
	203	Bahnbeamter im höheren Dienst	High-level railway official	A	1

Navigation	210	Seefahrer[b]	Sailor[b]	C	10
	211	Matrose, Leichterschiffer, Schiffsarbeiter	Seaman, lighterman, ship worker	C	823
	212	Bootsmann	Boatswain, petty officer	C	0
	213	Steuermann	Steersman, helmsman	B	172
	214	Schiffer, Kapitän	Skipper, ship's master	B	398
Machine construction	221	Maschinenarbeiter	Machine worker	C	18
	222	Maschinist	Machinist	B	135
	223	Maschinenmeister	Master machinist	B	2
Military	231	Soldat	Soldier	C	18
	232	Unteroffizier	Non-commissioned officer	C	15
	233	Offizier	Commissioned officer	B	0
	234	Hauptmann	Captain	B	6
					1,633
3) Crafts and arts					
Construction	300	Maurer, Steinmetz, Zimmermann, Maler u. a.[b]	Mason, stone mason, carpenter, painter etc.[b]	B	820
	301	- Gehilfe, Lehrling, Bauarbeiter	- Apprentice, building labourer	C	5
	302	- Geselle	- Journeyman	B	602
	303	- Meister	- Master	B	131
	304	Baumeister, -unternehmer	Master builder, building contractor	A	38
Shipbuilding	310	Schiffszimmermann[b]	Ship carpenter[b]	B	296
	311	- Gehilfe, Lehrling, Werftarbeiter	- Apprentice, shipyard worker	C	0
	312	- Geselle	- Journeyman	B	176
	313	- Meister	- Master	B	4
	314	Schiffsbaumeister	Master shipwright	A	22
Wood, metal	320	Tischler, Böttcher, Instrumentenmacher, Schmied u. a.[b]	Joiner, cooper, instrument maker, smith etc.[b]	B	585
	321	- Gehilfe, Lehrling	- Apprentice	C	0
	322	- Geselle	- Journeyman	B	152
	323	- Meister	- Master	B	214
Textiles, clothing, leather	330	Schneider, Weber, Schuhmacher, Riemer u. a.[b]	Tailor, weaver, shoemaker, leather worker etc.[b]	B	676
	331	- Gehilfe, Lehrling	- Apprentice	C	0
	332	- Geselle	- Journeyman	B	129
	333	- Meister	- Master	B	382
Other materials	340	Sonstiger Handwerker[b]	Other craftsman[b]	B	94
	341	- Gehilfe, Lehrling	- Apprentice	C	0
	342	- Geselle	- Journeyman	B	46
	343	- Meister	- Master	B	41
Hairdressing	350	Frisör, Barbier, Perückenmacher[b]	Hairdresser, barber, wig maker[b]	B	21
	351	- Gehilfe, Lehrling	- Apprentice	C	0
	352	- Geselle	- Journeyman	B	0
	353	- Meister	- Master	B	2
Nutrition	360	Bäcker, Müller, Schlachter, Zigarrenmacher etc.[b]	Baker, miller, slaughterer, cigar maker etc.[b]	B	311
	361	- Gehilfe, Lehrling	- Apprentice	C	4
	362	- Geselle	- Journeyman	B	32
	363	- Meister	- Master	B	116
Beer	370	Bierbrauer[b]	Brewer[b]	A	8
	371	Brauerlehrling	Apprentice brewer	C	0
	372	Kleinbierbrauer, Schoppenbrauer	Micro-brewer	B	9
	373	Braumeister	Master brewer	A	4
	374	Brauereibesitzer	Brewery owner	A	0

Print, Press, Photography	380	Drucker, Redakteur, Fotograf, Lithograf[b]	Printer, editor, photographer, lithographer[b]	B	72
	381	Buchdruckergehilfe	Apprentice book printer	C	12
	382	Buchdruckergeselle	Journeyman book printer	B	2
	383	Buchdruckereifaktor	Printing factor	B	4
	384	Buchdruckereibesitzer	Printing house owner	B	4
Arts	391	Musiker, Bildhauer, Künstler, Schauspieler, Gymnastiker	Musician, sculptor, artist, actor, gymnast	C	110
	392	Meister oder Lehrer der Künste	Master or teacher of arts	B	21
	393	Musik- oder Schauspieldirektor	Musical or theatrical director	A	0
	394	Städtischer oder königlicher Musikdirektor	City or royal musical director	A	0
					5,145
4) Unskilled work and manufacturing					
Unskilled work	400	Arbeitsmann[b]	Labourer[b]	C	4,460
	401	Diener, Tagelöhner	Hired servant, day labourer	C	187
	402	Träger	Porter	C	72
	403	Vorarbeiter	Foreman	B	0
Factory work	411	Fabrikarbeiter	Factory worker	C	87
	412	Fabrikgeselle	Factory journeyman	B	3
	413	Werkmeister	Master workman	B	15
	414	Fabrikant, Fabrikdirektor	Manufacturer, factory owner	A	21
					4,845
5) Agriculture					
Land use and holding	501	Ackersmann, Pächter	Field worker	C	263
	502	Büdner, Häusler, Ökonom	Cottager	B	16
	503	Eigentümer	Proprietor	A	3
	504	Gutsbesitzer	Landowner	A	7
Grain crop	511	Schnitter	Harvester	C	0
	512	Vorschnitter	Harvester foreman	B	0
Gardening	520	Gärtner[b]	Gardener[b]	B	66
	521	Gärtnergehilfe, Gartenarbeiter	Apprentice gardener, garden worker	C	0
	522	Handels-, Landschafts-, Gemüse-, Obst-, Kunstgärtner	Market gardener, landscaper, horticulturist, fruit grower	B	19
	523	Gärtnereibesitzer	Gardening shop owner	B	5
Distillery	531	Brennerknecht	Distiller's servant	C	0
	532	Branntweinbrenner	Distiller	C	23
	533	Brennereiverwalter	Distillery administrator	B	0
Fishing	540	Fischer[b]	Fisherman[b]	C	97
Animal breeding	551	Stallknecht	Groom	C	7
	552	Hirte, Schäfer, Bereiter	Shepherd, horsebreaker	C	5
	553	Reitlehrer	Riding instructor	B	1
Forestry, Hunting	561	Forstarbeiter, Kammerjäger	Forestry worker, exterminator	C	4
	562	Förster, Jäger	Forester, hunter	B	2
	563	Forstmeister, Jägermeister	Master forester, professional hunter	B	0
					518
6) Unknown or not employed					
	600	Keine Angabe	Not specified	C	35
	610	Pensionär, Rentier, Privatmann	Pensionary	A	35
	620	Invalide	Disabled person	C	14
	630	Schüler	Pupil	C	1
	640	Vater unbekannt[c]	Father unknown[c]	C	2,652
					2,737
Total					16,880

Notes:

[a] This classification was done using all transcribed entries of live births, stillbirths and infant deaths recorded in the baptismal and burial registers of St. Jakobi. As a result of limiting the data to the live births born in the periods 1815–1836 and 1859–1882, some categories show no numbers in this table.

[b] General category without specified status/rank/position within occupational group.

[c] This does not include unmarried fathers whose (assumed) identity (N=352) and job (N=321) were specified. For the survival analyses, they were also put into social class C, though.

Supplementary figures and tables

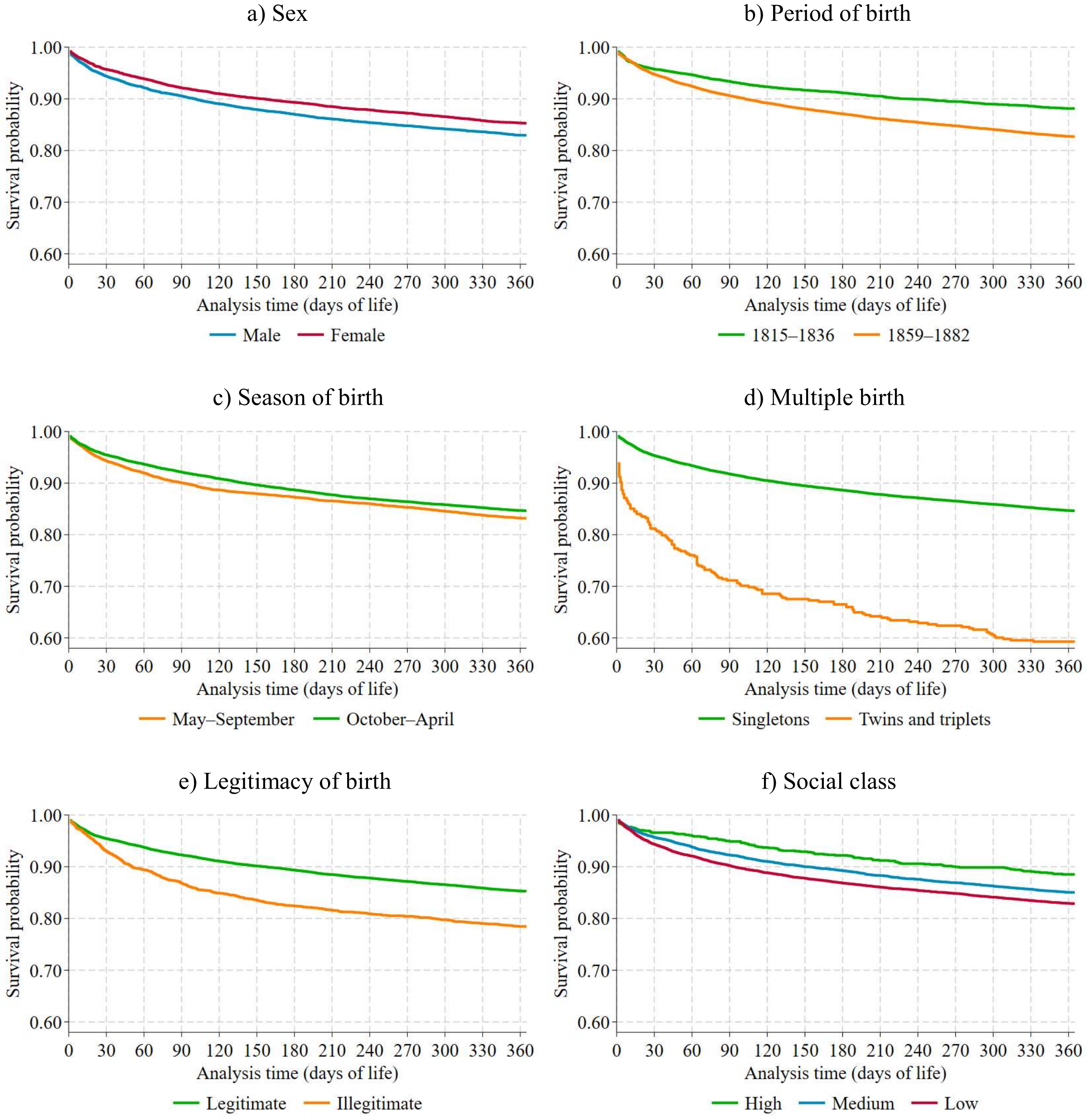

Fig. S1 Kaplan-Meier survival curves by sex, period, season, legitimacy, and social class in Rostock, St. Jakobi parish, 1815–1836 and 1859–1882.

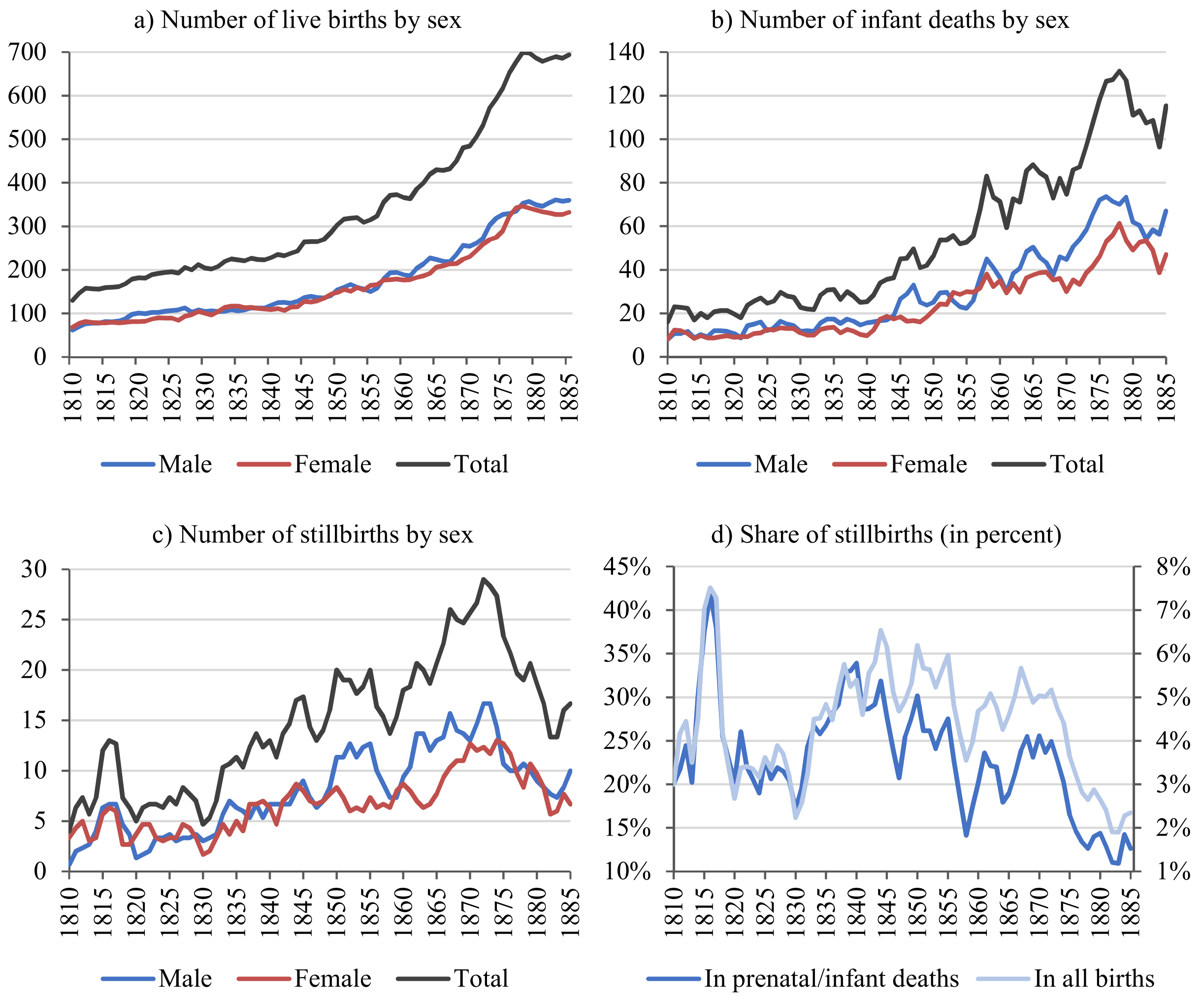

Fig. S2 Number of live births (a), infant deaths (b), and stillbirths (c) by sex, and share of stillbirths (d) in prenatal/infant deaths (left scale) and in all births (right scale) in Rostock, calendar years 1810–1885 (three-year moving average). Note: Prenatal/infant deaths is the sum of stillbirths and infant deaths. To show these long-term trends, we counted the frequencies of live births and stillbirths by sex for those years that were not transcribed.

Table S3 Number of live births by father's social class (with unmarried fathers in a separate group) and HISCLASS-5 in Rostock, 1815–1836 and 1859–1882.

		Social class				Total
		High	Medium	Low	Unmarried	
HISCLASS	Elite	529	0	0	11	540
	Lower middle class	532	1,520	192	25	2,269
	Skilled workers	11	4,786	1,227	214	6,238
	Self-employed farmers and fishermen	8	100	97	11	216
	Unskilled workers and farm workers	0	0	4,772	60	4,832
	No code in HISCLASS	35	0	67	2,683	2,785
Total		1,115	6,406	6,355	3,004	16,880
